# Ageing with Parkinson’s: Identification of Personal Needs in the Northern Spanish Context

**DOI:** 10.3390/healthcare12040498

**Published:** 2024-02-19

**Authors:** Patricia Solís García, María Souto Serrano, Mª Ángeles Alcedo Rodríguez, Elsa Peña Suárez, Ignacio Pedrosa, Antonio León Aguado Diaz

**Affiliations:** 1 Faculty of Education, Universidad Internacional de La Rioja, 26006 Logroño, Spain; 2 Asturian Society of Rehabilitation Psychology, 33001 Oviedo, Spain; mariasouto83@hotmail.com; 3 Faculty of Psychology, Universidad de Oviedo, 33009 Oviedo, Spain; malcedo@uniovi.es (M.Á.A.R.); aaguado17@gmail.com (A.L.A.D.); 4 Department of Education of the Government of the Principality of Asturias, 33007 Oviedo, Spain; elsaps@educastur.org; 5 Information and Communication Technology Center, 33203 Gijón, Spain; npedrosa@cop.es

**Keywords:** clinical health psychology, health care, elder, quality of life, Parkinson’s disease

## Abstract

As individuals diagnosed with Parkinson’s disease enter older age, the myriad challenges and complications associated with this condition tend to escalate. Hence, there is a critical necessity to comprehensively discern the perceived needs of these individuals, along with their proposed remedies and essential support requisites. Additionally, understanding the perspectives of their families becomes imperative to formulate tailored interventions aimed at enhancing their overall development, progression, and quality of life. The study’s main objective is to assess the perceived needs of individuals with PD and their family members, propose necessary solutions, and suggest future perspectives. The study encompassed a cohort of 268 participants, comprising 179 individuals diagnosed with Parkinson’s disease and 89 of their relatives. A meticulously designed structured interview instrument consisting of 93 items was employed to assess various domains encompassing perceived needs, institutional support mechanisms, essential solutions, and future anticipations. Results: Statistically significant differences were found in health resources, social services resources, obstacles, solutions, and future outlook, with higher mean values from the relatives. Conclusions: The results highlight the most concerning needs in this context. Specifically, those needs related to health resources, social services resources, and future outlook present the greatest differences between the two subsamples, with the family members perceiving more needs. This alignment extended to both the categorization of unmet needs and the requisite solutions envisioned to address them. Suggested improvements include a sociosanitary strategy, stakeholder involvement, and prioritizing flexible home assistance to support older individuals with PD and their families.

## 1. Introduction

Parkinson’s disease (PD) presents a prevalent neurological disorder with a crude prevalence between 100 and 200 per 100,000 persons, expected to rise due to aging populations and increased life expectancy [1,2]. PD ranks as the second-most common progressive neurodegenerative disorder, often leading to substantial disability, discrimination, and stigma, impacting the overall quality of life (QoL) [3].

As individuals affected by PD age, the challenges posed by this illness also grow [4]. The lower prevalence and wide geographical dispersion can result in isolation and hinder social interaction among those affected [5].

Motor complications related to walking and posture control, fatigue, pain, psychiatric issues, sleep disturbances, and mood disorders significantly affect the lives of those with PD, highlighting multiple unmet needs [6,7,8,9,10,11,12]. In this regard, the perceived and unmet needs related to the motor profile in people with PD have not varied significantly since the 1980s [13]. On the other hand, researchers have also focused their interest on individuals who experienced a significant symptom burden and concerns at the early to middle disease stage [14], highlighting how relatively common non-motor neuropsychiatric features such as anxiety and depression can also lead to social isolation [15]. Other needs, including those of families, pertain to training and information related to health care associated with the disease, symptoms, how to recognize them, coping strategies, and necessary lifestyle changes [16]. Identifying and addressing these needs are closely tied to enhancing quality of life, where meeting all requirements determines a good quality of life [17,18,19].

Unfortunately, these needs often receive inadequate attention, and information on this subject is relatively scarce [20,21]. Therefore, there is a critical need to identify the perceived needs, propose solutions, and determine necessary measures and support, including those perceived by family members. Understanding the needs of individuals with PD is crucial for providing essential services and enhancing their progress and quality of life [22,23].

The repercussions of this disease extend beyond the affected individuals to impact their families significantly. The symptomatology’s dependency often positions families as the primary caregivers, experiencing their own substantial support needs [24]. However, if families lack the necessary assistance and professional services for patient care, they themselves may suffer considerably [25].

Projections suggest that families will increasingly struggle to meet these needs [26]. The burden on families, associated with halting work, loss of social connections, and increased caregiving time, restricts the possibility of keeping individuals within their familiar environments. Therefore, a reevaluation of efforts between families, private sectors, and the state becomes imperative to establish an effective care model. Furthermore, there is a pressing need for developing therapies and care plans that can address the multifaceted needs of PD, ultimately improving the lives of those affected and their families. Finally, tailored support plans must be designed to offer appropriate services and assistance according to specific needs [27].

Given this context and the lack of conclusive data [28], the study’s main objective is to assess the perceived needs of individuals with PD and their family members, propose necessary solutions, and suggest future perspectives. From this overall objective, the following research questions arise: (I) What are the most prominent perceived needs of individuals diagnosed with PD and their family members, according to the assessment results? (II) What solutions are proposed as necessary to address the identified needs during the evaluation of individuals with PD and their family members? (III) What future perspectives are suggested by the participants regarding the needs and proposed solutions in the context of PD?

The following initial hypotheses are proposed: (I) Perceived needs of individuals with PD will mainly be related to health, economic and healthcare resources, dependence, home assistance, and the existence of barriers. (II) Proposed solutions, in relation to this first hypothesis, are directly related to health, lack of or difficulty with economic resources, the development of dependence in terms of care, specialization of home assistance, and the control of barriers. (III) Support will be needed, to be developed in the institutional, economic, healthcare, family, and social domains. (IV) There will be no significant differences in perceived needs between individuals with PD and their family members.

By gathering this information, the study aims to pinpoint the most crucial areas and differences between individuals with PD and their relatives regarding identifying these needs. This study aims to provide a valuable contribution by delving into a comprehensive understanding of the perceived needs of individuals diagnosed with PD and their families as they face the challenges associated with aging. This alignment underscores the importance of considering both perspectives in order to formulate tailored interventions to enhance overall development, progression, and quality of life for those affected by PD in older age. To achieve this, the study assesses the needs of the primary stakeholders in this disease—the aging individuals with PD and their families. Only by understanding their needs can resources be effectively planned to enhance individual autonomy and quality of life for both individuals with PD and their families.

## 2. Materials and Methods

### 2.1. Participants

The study included 268 participants categorized into two groups: individuals diagnosed with PD and their respective relatives. All participants resided in Spain, with the majority (82.68%) hailing from the Principality of Asturias.

The PD group consisted of 179 participants, aged between 45 and 92 years (M = 72.40; SD = 9.40), with 54.75% being female. Among them, 65.37% lived in family settings, 15.08% in supportive living facilities, and 16.2% lived alone.

The group of relatives reporting on the needs of PD individuals comprised 89 participants (53.93% women). Predominantly, they were identified as sons/daughters (38.20%) or spouses (37.80%), with ages ranging from 46 to 91 years (M = 72.01; SD = 9.96). Note that there is a smaller number of individuals in the sample of family members compared to individuals with PD because many did not have a close relative willing to complete the interview.

### 2.2. Instrument

Needs evaluation was conducted using the Interview for Needs Assessment of Ageing People with Disabilities (INEAD), a well-established interview consisting of 93 items; this scale has been used in previous studies and has estimated reliability coefficient (alpha coefficient of 0.85) [20]. The personal interview is a semi-structured interview that combines closed-ended questions with multiple-choice alternatives, with a final open-ended interview. It gathers comprehensive information on descriptive variables related to disability and sociodemographic and environmental variables (personal data, interview completion data, clinical data, and information about living arrangements). Additionally, it assesses eight categories of needs: personal health, economic resources, health resources and social services resources, physical and social barriers, social support measures and networks, solutions required, and future outlook. Finally, an open-ended question is included for “other considerations to add”. On the other hand, the family interview is formally integrated into the final part of the personal interview and consists of 6 questions regarding the family members’ opinions about concerns and needs, solutions, institutional measures and support, and thoughts about the future of the person with Parkinson’s.

### 2.3. Data Analysis

Initially, normality was assessed using the Kolmogorov–Smirnov (K–S) test. Subsequently, non-parametric tests were employed to analyze perceived needs, solutions required, and future outlook. The effect size was estimated using Cohen’s (1988) criteria (η^2^) with values categorized as low (≤0.02), moderate (between 0.03 and 0.14), and large (>0.14). Independence tests (Χ^2^) were applied, and the phi coefficient measured associations between variables. IBM SPSS 20 for Windows facilitated all analyses, conducted at a CL = 95%.

### 2.4. Procedure

To gather sample data, we proceeded to contact all public and private services that cater to individuals with PD in the northern region of Spain. First, a list of potential candidate centers was developed, and the necessary institutional contacts were established with the entities involved to inform them about the study and coordinate meetings with the individuals concerned. This included more than 100 associations, residential centers, healthcare facilities, support services, and rehabilitation services. Professionals and representatives from these centers and institutions disseminated information about the research among their associates through letters or phone calls. In all cases, prior consent was requested for participation. Once individuals expressed their willingness to collaborate, personal interviews were scheduled. All participants received information about the study and its objectives, and confidentiality and anonymous use of the information for research purposes were guaranteed. The recruitment of the sample was carried out in compliance with the regulations governing the protection of personal data (LOPD 15/1999).

Given the mobility and accessibility challenges faced by this group, the interviews were conducted individually in accessible premises of the associations or by visiting the home or residence of the evaluated person. The completion of each interview took approximately 60–75 min. The interviews were administered individually to individuals with Parkinson’s disease and their families by the interviewing team, which had received prior information and training. In cases where participants faced communication or understanding limitations, family members or caregivers familiar with the individual’s needs provided assistance. In those cases where the interview could not be completed or data were missing, the entire interview was discarded.

Ethical considerations: The principles for conducting research contained in the Declaration of Helsinki were respected. Although the study involves the participation of human subjects, no experimentation has been conducted on them and confidentiality and voluntariness of data have been ensured, complying with articles 40 and 45 of the Deontological Code of Psychology in Spain, which can be cited as national legislation safeguarding the rights and duties of assessment within the field of Psychology, as well as with the guidelines of the international document ‘Guide for the Assessment Process’. All participants were informed of the research and its objectives. Permission and informed consent were collected from all participants. Removing names and using identifying codes that the researchers did not know guaranteed confidentiality and anonymity.

## 3. Results

As the data did not fit a normal distribution, the Mann–Whitney U test was used to examine the statistical differences between both subsamples. Statistically significant differences were found in health resources, social services resources, obstacles, solutions, and future outlook, with higher mean values from the relatives (Table 1). In order to avoid possible sample size influences on these results, the effect size (*p* < 0.05) was estimated, giving overall large results (ranging from 0.24 to 0.90).

The factor of “personal health” encompasses concerns related to overall health status, dependence, the presence of pain, daily personal care, and difficulties in medication management, among others. The “economic resources” factor aggregates concerns related to pensions, family economic situation, economic independence, grants, and work disability. The “health resources” area encompasses concerns related to treatment and physiotherapy, improved healthcare facilities, hospital healthcare, quality of healthcare, and lack of information about these resources, among others. Concerns related to “social services resources” address home assistance, improved social facilities, lack of information about these resources, support services, and leisure time, among others. The “physical and social barriers” area addresses concerns about architectural barriers, transportation ease, accessibility to public buildings, social barriers, societal acceptance, etc. In the “social support and network” area, support from municipalities, equal opportunities, training activities, and environmental support, among others, are addressed. Regarding “solutions required”, all those related to previous needs are addressed, and as for “future outlook”, thoughts regarding living with quality of life, personal autonomy, loneliness, boredom, etc., are considered.

Pearson Chi-squared tests were then performed between the areas and categories that were statistically significant in the previous analysis in order to specify in which items the differences were found.

For the existence of physical and social obstacles, there were only significant differences in “technical assistance which aids mobility and access”. However, this difference disappeared once Yates’ correction (*p* > 0.05) was applied.

In terms of factors related to health and social services resources and future outlook, which demonstrated statistically significant differences, all of the items except “rely on physiotherapy treatment” suggested more concern on the part of the families (Table 2).

Similar results were found with respect to the types of solutions needed (Table 3). Again, there were statistically significant differences between the two subsamples, with the family members requiring more solutions, except for the availability of help for personal daily care, having more and better health facilities, having more specialist health professionals, more transport facilities, promotion of adapted living spaces, and increased resources from patient associations and community support.

Looking at the Phi coefficient, it is worth mentioning the concern shown by the relatives regarding the difficulty the person with PD has in achieving an independent future with no problems, which is related to those solutions linked to increased family support and the need for those people with PD to have more resources from patient associations.

## 4. Discussion

The study’s main objective is to assess the perceived needs of individuals with PD and their family members, propose necessary solutions, and suggest future perspectives, since any proposed course of action must stem from a thorough study and analysis of the reality characterizing this aging process. In general, the profile of older individuals with PD who participated in this research is quite heterogeneous.

The evaluation instrument used to discover the needs of ageing people with PD incorporates a view of systems which encompasses the multiple settings that have an impact on the person (e.g., the family) while at the same time including the participation of the subject with PD themselves in the evaluation of their needs. The results are in line with previous research, confirming that more than half of the population with PD live with their family, who are the main caregivers, combining that care with their support needs [29,30]. For that reason, it is crucial to include these carers’ perspectives in this study, as it should never be forgotten that a person’s quality of life cannot be isolated from the care they receive or the people who manage that care [31].

The results highlight the most concerning needs in this context. Specifically, those needs related to health resources, social services resources, and future outlook present the greatest differences between the two subsamples, with the family members perceiving more needs. The main concerns about health resources revolve around the quality of the health care itself and treatment. These results are not surprising, given the frequent motor complications in PD, which have a marked impact on these people’s quality of life [32]. Faced with this, it seems necessary to reinforce strategic health plans to make the necessary support available and to achieve the best outcomes possible [33].

Regarding social services resources, the participants demonstrated particular concern about having help in the home and having good quality social help. The family members more often indicated these issues. These results suggest that, despite the undeniable preventive and family-supporting nature of social services, current access to professional care is insufficient [34]. A possible explanation for this, it should be noted, is that these types of services are provided by public administrations to a small number of homes, making it extremely expensive for many families in the current socioeconomic situation. When developing interventions, the development of this type of resource would reduce the burden on carers, making it possible for people with PD to stay in the family environment, as families are not capable of providing total care for those with PD, especially in the later stages of the illness.

When considering the future, the biggest worries are about autonomy, loneliness and independence of those with PD. Staying within their individual, family and social environment is a facilitating factor in satisfaction and quality of life [30]. However, from the carer’s perspective, there are more needs in this category. This is logical, as most of the care this kind of health problem requires can bring with it rather negative effects; one notable example is high stress [20,35]. The only way to alleviate these needs and prevent future problems is to make resources available to those caregivers, which compensate for lack of functioning, avoid total dependency on another person, and facilitate social contact [36]. Nonetheless, research suggests a lack of resources and support for families, who will find that their capacity to respond to the needs of those with DP diminishes as they get older [37].

The results demonstrate a significant requirement for solutions for a similar number of perceived needs. Chief among the solutions required by those with PD are increased patient association resources (42.50%) and the need to have more and better health facilities (33.50%). The care and services provided to those with PD and their families by these various associations are fundamental, bearing the brunt of specialist care, which is not simple as it is not managed by public health and social care systems [38]. In the face of this problem, some solutions should be geared towards those approaches that the older adults suggest, such as having more specialized professionals (29.60%), and availability of daily personal care (27.90%), ensuring the maintenance of their quality of life [39].

The relatives requested more solutions to respond to their perceived needs. They proposed things such as increased family support (56.20%), having more and better information about the health of the person with PD (42.70%), leisure and free time services (48.30%), and coordination between various administrations (47.20%). It is important to highlight the need for these services because if the right level of family involvement is not achieved, it could lead to serious pathologies such as anxiety, stress [35], or burnout syndrome. These issues, which put the emotional well-being of carers at risk, require there to be quality social support networks which provide the families with the support they need [40].

In summary, this research provides a holistic view of needs evaluation in the area of PD thanks to the participation of the two principal agents involved: the ageing person with PD and the family [23]. Regarding the coincidences and discrepancies observed in the comparative analysis regarding perceived needs and concerns in general, there is a certain harmony between individuals and families. Both groups prioritize personal health issues, and it is worth noting that PD presents a sequence of health problems that manifest as a consequence of the progression of the disease and disability [34]. To such an extent, both groups identify assistance for daily personal care and support for informal caregivers as a solution.

Although the family members tended to indicate more needs, they agree with the people with PD in terms of the type of needs which are not being met and the solutions they require, sharing the demand for more and better services in which quality is the prime factor. It indicates a call for the implementation of activities and solutions appropriate to these unmet needs so that those with PD can enjoy a sufficient quality of life following diagnosis [41]. To that end, it is crucial to provide good quality services, as that is the best guarantee of respecting these people’s rights and meeting their needs [42]. Firstly, the study is based on the perception of needs from the two fundamental agents, but it should be noted that the sample size is limited, and the study focuses on a specific geographical area. Additionally, there has not been an in-depth analysis regarding the influence of sociodemographic variables on the perception of needs in individuals with PD. It is important to acknowledge these limitations to ensure a nuanced interpretation of the findings. Furthermore, a more comprehensive view of the subject could be achieved by including the opinions of professionals working in this field. Secondly, it would be interesting to design a longitudinal study to provide a detailed view of the development of needs as a person ages, allowing the definition of intervention measures appropriate to individual characteristics.

Considering the discussed data, some improvement proposals are suggested, encompassing various areas, and outlined in the following intervention guidelines. Firstly, there should be an encouragement for the implementation of a sociosanitary strategy involving all relevant stakeholders to integrate the available resources in addressing the needs of older individuals with PD and their families. Additionally, there should be a prioritization of home assistance tailored to the needs of users and the family unit, thus being flexible in terms of schedules and tasks, thereby promoting the continued residence of the older person with PD within their family environment.

## 5. Conclusions

Key findings included concerns regarding health resources, social services resources, and future outlook, with family members expressing more significant needs. Health resource concerns pertained to the quality of healthcare and treatment, while social services resource concerns focused on in-home assistance and the quality of social help. For the future outlook, the main worries were related to autonomy, loneliness, and independence, particularly from the perspective of family members.

Solutions required included a need for increased patient association resources and better health facilities from the perspective of individuals with PD. Family members sought more solutions, such as enhanced family support, better health information, increased leisure and free time services, and improved coordination between administrations.

In conclusion, this comprehensive study emphasized the importance of considering the needs of both individuals with PD and their family members to enhance the quality of life and wellbeing of those affected. While family members expressed more concerns and sought more solutions, their alignment on unmet needs highlights the necessity for increased and higher-quality services to support individuals with PD as well as their caregivers. These results underscore the importance of respecting patients’ rights and addressing their needs. The findings provide valuable insights for developing targeted intervention measures and warrant further research, potentially involving professionals in the field, to gain a more comprehensive understanding of the needs and experiences of those living with PD and their families. Additionally, a longitudinal study could shed light on the evolution of needs as individuals age, guiding the development of personalized intervention strategies.

## Figures and Tables

**Table 1:** Results of the Mann–Whitney U test on perceived needs, solutions required, and future outlook.

	Subsample	Mean Rank	*χ*^2^ (df)	Asymp. Sig.	η^2^
Personal health	PDP	132.65	0.33 (1)	>0.05	-
	Relatives	138.23			
Economic resources	PDP	129.35	2.87 (1)	>0.05	-
	Relatives	144.87			
Health resources	PDP	127.99	3.96 (1)	<0.05	0.24
	Relatives	147.59			
Social services resources	PDP	122.07	14.80 (1)	<0.05	0.90
	Relatives	159.49			
Physical and social barriers	PDP	127.65	4.56 (1)	<0.05	0.28
	Relatives	148.28			
Social support measures and network	PDP	129.85	2.12 (1)	>0.05	-
	Relatives	143.85			
Solutions required	PDP	122.20	13.63 (1)	<0.05	0.83
	Relatives	159.23			
Future outlook	PDP	128.10	3.97 (1)	<0.05	0.24
	Relatives	147.37			

Note. PDP: people with Parkinson’s disease; df = degrees of freedom; η^2^ = estimated effect size.

**Table 2:** Statistically significant types of health resources, social services resources, and future outlook.

			Yes (%)	*χ*^2^ (df)	*φ*	Asymp. Sig.
Health Resources	Difficulties in attending treatment	PDP	10.6	10.62 (1)	−0.210	<0.01
		Relatives	27.0			
	Have support appliances and prosthetics	PDP	10.6	30.64 (1)	−0.348	<0.01
		Relatives	40.4			
	Have physiotherapeutic treatment	PDP	33.5	5.35 (1)	0.150	<0.05
		Relatives	19.1			
	Information on health and care resources	PDP	16.2	6.36 (1)	−0.164	<0.05
		Relatives	30.3			
	Quality of healthcare	PDP	25.1	4.27 (1)	−0.135	<0.05
		Relatives	38.2			
Social services resources	Have help in the home	PDP	33.5	4.89 (1)	−0.143	<0.05
		Relatives	48.3			
	Appropriate quality of social services attendance	PDP	16.2	12.09 (1)	−0.222	<0.01
		Relatives	36.0			
Future outlook	Face a solitary future	PDP	10.1	40.63 (1)	−0.399	<0.01
		Relatives	44.9			
	A future living independently	PDP	10.1	59.31 (1)	−0.480	<0.01
		Relatives	53.9			
	Face an uncertain, worrying future	PDP	17.3	11.70 (1)	−0.218	<0.01
		Relatives	37.1			
	Face the future with no problems	PDP	9.5	44.75 (1)	−0.418	<0.01
		Relatives	46.1			
	Face the future with personal autonomy	PDP	19.0	14.42 (1)	−0.241	<0.01
		Relatives	41.6			
	Have assurance about the future	PDP	19.0	11.81 (1)	−0.219	<0.01
		Relatives	39.3			

Note. Yes % = percentage of people with Parkinson’s disease (PDP) and relatives who mention this indicator; df = degrees of freedom; *φ* = phi coefficient.

**Table 3:** Statistically significant solutions are required.

		Yes (%)	*χ*^2^ (df)	*φ*	Asymp. Sig.
Availability of help for personal daily care	PDP	27.9	6.19 (1)	0.161	<0.05
	Relatives	13.5			
Increased family help	PDP	12.8	54.15 (1)	−0.458	<0.01
	Relatives	56.2			
Have more and better health installations	PDP	33.5	4.47 (1)	0.138	<0.05
	Relatives	20.2			
Retire earlier	PDP	5.6	17.13 (1)	−0.265	<0.01
	Relatives	23.6			
Have more specialized professionals	PDP	29.6	7.56 (1)	0.177	<0.01
	Relatives	13.5			
Improved prosthetics support	PDP	10.6	13.42 (1)	−0.234	<0.01
	Relatives	29.2			
Have more and better information about health	PDP	21.8	11.69 (1)	−0.218	<0.01
	Relatives	42.7			
Availability of legal and administrative support	PDP	11.7	7.63 (1)	−0.179	<0.01
	Relatives	25.8			
Better quality of health care	PDP	19.6	11.04 (1)	−0.212	<0.01
	Relatives	39.3			
Availability of support services for leisure and free time	PDP	19.6	22.46 (1)	−0.298	<0.01
	Relatives	48.3			
Societal acceptance	PDP	12.8	21.33 (1)	−0.292	<0.01
	Relatives	38.2			
Acceptance by the family	PDP	8.4	29.38 (1)	−0.342	<0.01
	Relatives	36.0			
More transport facilities	PDP	30.7	16.21 (1)	0.255	<.01
	Relatives	7.9			
Promote adapted living arrangements	PDP	21.8	8.58 (1)	0.190	<0.01
	Relatives	6.7			
Support for informal carers	PDP	23.5	4.04 (1)	−0.132	<0.05
	Relatives	36.0			
Coordination between different administrations	PDP	25.7	11.50 (1)	−0.216	<0.01
	Relatives	47.2			
Increased resources for associations	PDP	42.5	39.12 (1)	0.391	<0.01
	Relatives	4.5			
Community support	PDP	22.9	15.14 (1)	0.248	<0.01
	Relatives	3.4			

Note. Yes % = percentage of people with Parkinson’s disease (PDP) and relatives who mention this indicator; df = degrees of freedom; *φ* = phi coefficient.

## Data Availability

The data presented in this study are available on request from the corresponding author. The data are not publicly available due to privacy considerations.
